# Decade-long protection of the mumps vaccine: Insights from a large-scale serological study

**DOI:** 10.1371/journal.pntd.0013125

**Published:** 2025-06-03

**Authors:** Zixia Qian, Yueling Chen, Lilian Zeng, Chumin Liang, Weizhao Lin, Can Xiong, Xinxin Li, Yingyin Deng, Liang Chen, Ying Yang, Limei Sun, Jianfeng He, Jiufeng Sun

**Affiliations:** 1 School of Public Health, Guangdong Pharmaceutical University, Guangzhou, Guangdong, China; 2 Guangdong Provincial Institute of Public Health, Guangdong Provincial Center for Disease Control and Prevention, Guangzhou, Guangdong, China; 3 School of Public Health, Southern Medical University, Guangzhou, Guangdong, China; 4 Department of Public Health and Preventive Medicine, Jinan University, Guangzhou, Guangdong, China; 5 School of Public Health, Sun Yat-Sen University, Guangzhou, Guangdong, China; 6 Guangdong Workstation for Emerging Infectious Disease Control and Prevention, Guangdong Provincial Key Laboratory of Pathogen Detection for Emerging Infectious Disease Response, Guangdong Provincial Center for Disease Control and Prevention, Guangzhou, Guangdong, China; The University of Kansas, UNITED STATES OF AMERICA

## Abstract

**Background:**

Serological surveys of mumps are important for estimating susceptibility in the population and for evaluating the effectiveness of current vaccination strategies.

**Methods:**

A cross-sectional serological survey using an enzyme-linked immunosorbent assay was conducted on 5,147 participants in Guangdong, China, to evaluate the immunological effects of 2 doses of measles-mumps-rubella vaccine. In accordance with the instructions of the ELISA kit, the final readings represent the anti-mumps antibody titers, which are expressed in “NovaTec units”.

**Results:**

Of the 5,147 participants, 3,888 were positive for mumps IgG antibodies, with a seroprevalence of 75.54% (*95% CI*: 74.34%-76.71%). For each age group, the mumps IgG seroprevalence rates were 74.40%, 89.02%, 85.58%, 68.60%, 69.28%, 78.42%, and 80.63% for those <8 months, 8 months-2 years, 3–5 years, 6–17 years, 18–39 years, 40–59 years, and>=60 years, respectively. In terms of the percentage decreases in anti-mumps antibody titers, in the population receiving the 1-dose vaccine, there was a mean decrease of 2.06% per year. In the population receiving 2 doses of the vaccine, the mean annual decreases were 10.33% and reached protective thresholds of approximately 12.3 years.

**Conclusion:**

The high mumps seroprevalence in the unvaccinated population revealed neglected hidden mumps infections. A time-lapse assay of IgG antibodies indicated that the mumps vaccine provided protection for one decade, which highlights that booster vaccinations may be needed in adults.

## Author summary

Mumps is an inflammatory disease caused by the mumps virus. High-risk groups include children and adolescents, which may be related to a weaker immune system or ineffective vaccination. Serological surveillance of mumps is key to estimating population susceptibility and evaluating the effectiveness of the current vaccination strategy. China has adjusted a two-dose MMR program for approximately four years, but there is limited research on the immunological effects of the two-dose MMR on mumps. In this study, we used the enzyme-linked immunosorbent assay to measure the levels of anti-mumps IgG antibodies in the serum of healthy population participants in China, and analyzed the mumps seroprevalence between different groups. We found a declining trend in the healthy population after two doses of vaccination. The findings in this study will guide the reform of public health strategies for mumps control and prevention, as well as the adaptation of vaccination program.

## Introduction

Mumps is an acute respiratory infection that is caused by the mumps virus (MuV), a single-stranded, enveloped RNA virus belonging to the family *Paramyxoviridae*. Upon infection, the virus causes pain and swelling in the parotid salivary glands. To date, humans have been identified as unique natural hosts of MuV. Mumps is a highly contagious disease that typically manifests in children and adolescents and presents with symptoms such as headache, fever, and general malaise. Mumps also occurs in adults, with the potential for serious complications such as testicular inflammation, meningitis, deafness, and permanent nerve damage [1].

The World Health Organization (WHO) reported that the number of mumps cases reported globally in 2022 was 380,338, with an increase of 138,932 cases compared with 2021 [2]. The WHO recommended that countries with mature and effective childhood vaccination programs, which maintain high coverage rates for measles and rubella, consider incorporating mumps vaccinations into their routine immunization schedules and prioritize reducing the incidence of mumps as a public health goal [3]. By the end of 2022, the mumps vaccine had been fully rolled out in 123 member countries worldwide [4]. The continuous advancement of immunization programs in various countries has resulted in the effective control of mumps [5], with a concomitant decline in the global burden due to mumps [6]. However, outbreaks of mumps cases still occur in populations of countries in which mumps vaccines have been administered. This may be attributed to the breakthrough infections caused by the weakening of the immunity induced by the vaccine or the emergence of MuV genotypes that are not included in commercial vaccines [7]. Nevertheless, vaccination remains the primary strategy for the prevention of mumps, and individuals who have been vaccinated have a lower risk of developing complications compared to those who have not been vaccinated [8–10].

Since 1989, mumps has been classified as a legally reportable infectious disease in China and managed as a category C infectious disease [11]. In 2008, China expanded National Immunization Program (NIP) with the introduction of the measles-rubella vaccine (MR) for infants eight months old, followed by the measles-mumps-rubella vaccine (MMR) for infants 18–24 months old [12]. At the beginning of 2020, 8- and 18- months old immunization procedures were implemented with 2 doses of MMR [13]. The age-appropriate population refers to individuals who meet the age requirements for vaccination, especially children and adolescents who are required to receive routine vaccinations according to the NIP. As of 2023, the age-appropriate population for vaccination is concentrated in the 0–16 year age group, and the population for 2-dose vaccine group is mainly concentrated around the age of 0–3 years in our study. At present, research on sequential administration of MMR in China is relatively limited. Although China has included mumps in the national immunization program for more than a decade, the incidence remains high among children [14]. The national annual incidence rate of mumps was maintained at 12.84/100,000 to 35.59/100,000 from 2008–2018, with a peak level of more than 300,000 reported cases in 2019 [15]. The number of cases in 2020 slightly decreased, with 129,120 cases and 2 deaths and an incidence rate of 10.06/100,000 [16]. In a meta-analysis, Li et al. conducted a comprehensive assessment of the mumps IgG antibody levels in healthy individuals. A total of 97,034 healthy individuals from 26 administrative regions across China were included. The results revealed that the endemic prevalence of mumps was still high, with the lowest seroprevalence of 49.81% in Northeast China and the highest of 81.45% in North China [15]. Mumps still needs to be considered in China.

Therefore, it is necessary to strengthen the monitoring of the immune status of the population, which will favor adjusting the immunization strategy in a timely manner. Thus, the aim of this study was to assess mumps IgG antibodies in the healthy population of China, e.g., Guangdong Province, which has the largest population of more than one hundred million, and to evaluate the effectiveness of a 2-dose mumps-containing component vaccine.

## Results

### Demographic characteristics

A total of 5,147 samples from the participating individuals were involved and tested from 2021 to 2023, and the sample numbers in each year were 1,520 (29.53%), 1,927 (37.44%) and 1,700 (33.03%), respectively (S1 Data). The ages ranged from 0 to 100 years, with a mean of 32.12 years. The age distribution revealed that the 18–39 year group had the greatest number of participants, with 1,859 (36.12%) participants. Males accounted for 53.18%, and females accounted for 46.82%. With respect to geography, 2,505 cases (48.67%) were from Guangzhou, and 2,642 cases (51.33%) were from Heyuan. Among the age-appropriate population (0–16years), 842 individuals (61.06%) were confirmed as having received 1 dose or 2 doses of vaccination in the vaccination system, and 537 individuals (38.94%) were of unknown vaccination status or unvaccinated (Table 1).

**Table 1:** Baseline characteristics of study participants.

Characteristics		Number(n = 5147)	percentage (%)
Gender	Male	2737	53.18
	Female	2410	46.82
Region	Heyuan	2642	51.33
	Guangzhou	2505	48.67
Age	<8ms^a^	418	8.12
	8ms ~ 2ys^b^	82	1.59
	3 ~ 5 ys	617	11.99
	6 ~ 17 ys	363	7.05
	18 ~ 39 ys	1,859	36.12
	40 ~ 59 ys	848	16.48
	>=60 ys	960	18.65
Year	2021	1520	29.53
	2022	1927	37.44
	2023	1700	33.03
Immunisation^c^	Yes^d^	842	61.06
	No^e^	537	38.94

^a^ms: months; ^b^ys: years; ^c^Immunisation: Referring to the vaccination history of the appropriate age group, specifically those aged 0–16 years, with a total number of 1,379. ^d^Yes: Including populations who received either 1 or 2 doses of vaccination; ^e^No: Including populations who have not been vaccinated or those with undisclosed information.

### Comparison of mumps IgG antibody levels

**Fig 1:**
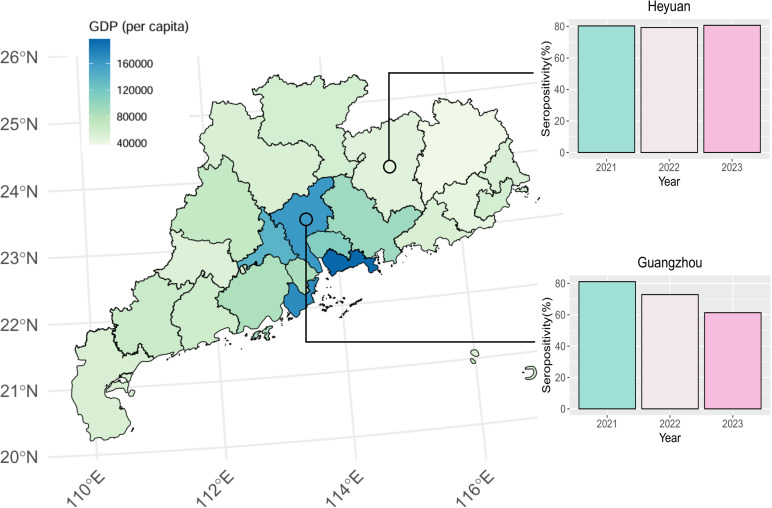
Heat map of per capita GDP in Guangdong province, and seroprevalence of Guangzhou and Heyuan. Source of basic map: Resource and Environmental Science Data Registration and Publishing System (https://www.resdc.cn/DOI/DOI.aspx?DOIid=121), License information: https://www.resdc.cn/NewsInfo.aspx?NewsID=9 [17].

**Fig 2:**
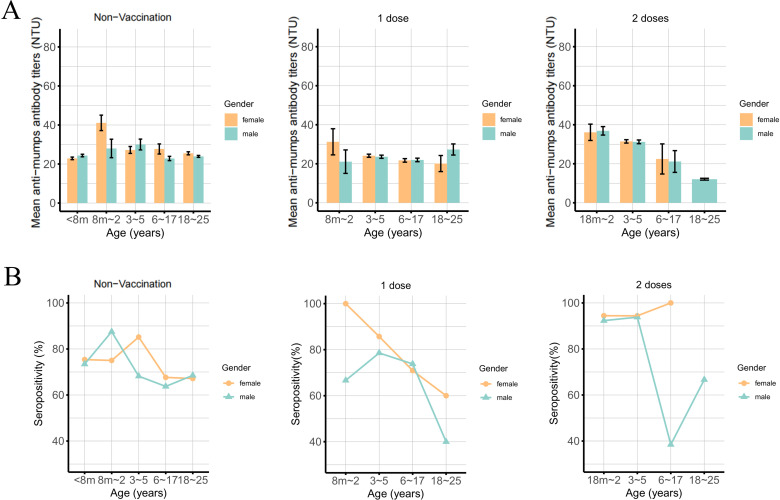
Histograms of mean anti-mumps antibody titers and seropositivity by vaccine dose and age (0-25 years). (A) Histograms of mean anti-mumps antibody titers with ages for 0, 1 and 2 doses vaccination (0-25 years), with a total number of 1,843. There were no females in the 18-25 years age group in the 2 doses population. Since the MMR was started at 8 months of age and the second dose was given at 18 months of age, data for the < 8 months of age group are missing for the 1 and 2 dose groups. (B) Line chart of seropositivity (%) for 0, 1 and 2 doses of inoculation in different age groups (0-25 years), with a total number of 1,843.

Among the 5,147 participants, 3,888 were positive for mumps IgG antibodies, with a seroprevalence rate of 75.54% (*95% CI:* 74.34%-76.71%). The total mumps IgG seroprevalence rates in males and females were 73.36% and 78.01% (*P *< 0.001), respectively. For each age group, the mumps IgG seroprevalence rates were 74.40%, 89.02%, 85.58%, 68.60%, 69.28%, 78.42%, and 80.63% (*P *< 0.001) for individuals <8 months, 8 months to 2 years, 3–5 years, 6–17 years, 18–39 years, 40–59 years, and>=60 years, respectively (Table 2). In the geography assay, the mumps IgG seroprevalence rates for healthy individuals in Guangzhou were 80.11%, 72.86%, and 61.33% from 2021-2023 as well as 80.35%, 79.37%, and 80.75% in Heyuan (Fig 1). Since 2001, Guangdong Province has gradually established a provincial immunization program information system. The age group with a history of vaccination is primarily concentrated between 0 and 25 years. Therefore, we further analyzed the effects of gender on the mumps seroprevalence rates and anti-mumps antibody titers under different vaccination protocols in the population aged 0–25 years (Fig 2). In the unvaccinated group, the anti-mumps antibody titers were greater in the older age group than in the younger age group, which are completely different from the results for the 1-dose- or 2-dose-vaccinated population (Fig 2A). However, in the population that received 1 dose or 2 doses, the mumps seroprevalence rates in females was higher than that in males in most age groups. Nevertheless, similar results were not observed in the unvaccinated group (Fig 2B).

**Table 2:** Number of samples tested, seropositivity and anti-mumps antibody titers in different groups.

Characteristics		Number	Mumps (n = 5147)					
			Seropositivity (n,%)	*χ* ^*2*^	*P-value*	anti-mumps antibody titers (NTUs)	*t/F*	*P-value*
Gender	Male	2737	2008(73.36)	14.95	<0.001	19.36	3.793	<0.001
	Female	2410	1880(78.01)			20.54		
Region	Heyuan	2642	2117(80.13)	61.88	<0.001	20.82	5.99	<0.001
	Guangzhou	2505	1771(70.70)			18.95		
Age	<8ms^a^	418	311(74.40)	108.08	<0.001	19.29	44.27	<0.001
	8ms ~ 2ys^b^	82	73(89.02)			32.61		
	3 ~ 5 ys	617	528(85.58)			24.18		
	6 ~ 17 ys	363	249(68.60)			17.76		
	18 ~ 39 ys	1,859	1288(69.28)			18.31		
	40 ~ 59 ys	848	665(78.42)			19.52		
	>=60 ys	960	774(80.63)			20.63		
Year	2021	1520	1226(80.66)	45.27	<0.001	21.12	21.72	<0.001
	2022	1927	1464(75.97)			20.15		
	2023	1700	1198(70.47)			18.57		
Immunisation^c^	Yes^d^	842	701 (83.25)	20.28	<0.001	23.35	32.66	<0.001
	No^e^	537	393(73.18)			19.66		

^a^ms: months; ^b^ys: years; ^c^Immunisation: Referring to the vaccination history of the appropriate age group (0–16 years), with a total number of 1,379. ^d^Yes: Including populations who received either 1 or 2 doses of vaccination; ^e^No: Including populations who have not been vaccinated or those with undisclosed information.

### Multifactorial log-binomial regression of mumps IgG seroprevalence

**Fig 3:**
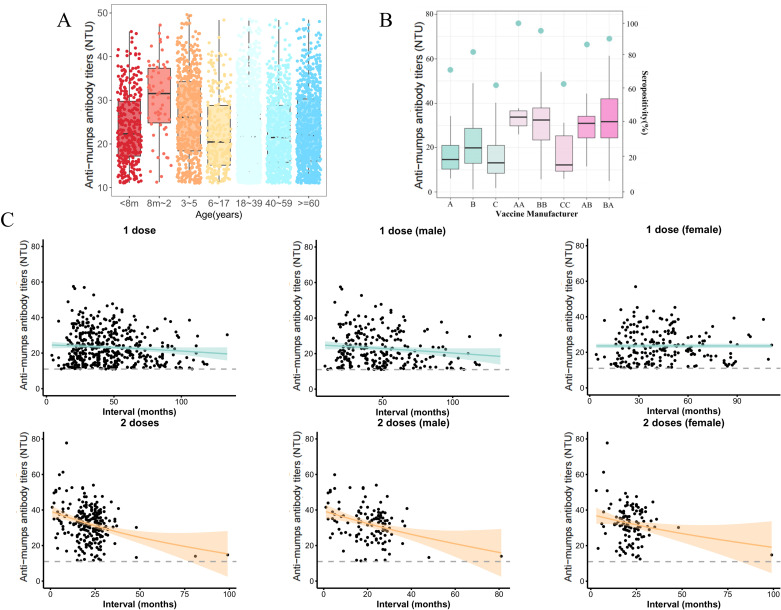
Anti-mumps antibody titers, Seropositivity, and IgG Decay. (A)The anti-mumps antibody titers (NTUs) with ages of 3,888 antibody-positive individuals. (B) The anti-mumps antibody titers (NTUs) and seropositivity (%) in different vaccine manufacturers. The Boxplot represents anti-mumps antibody titers, and the points represent seropositivity. X-axis: A, B, C represent Manufacturer A, B, and others for 1 dose vaccinations. AA, BB are 2 doses homologous vaccinations, CC stand for 2 doses others. AB and BA represent 2 doses heterologous vaccinations. (C) The decay tendency of anti-mumps IgG in all participants with a history of immunization. (the solid line represents the fitted declining trend of IgG antibodies against mumps, and the shaded area represents the *95%CI*).The green shaded area of the *95%CI* represents the group that received 1 dose, while the orange area represents the group that received 2 doses.The dashed line represents a threshold of 11 NTUs.

The mumps IgG seroprevalence rate was greater in females than in males (*OR*=1.28, *95% CI:* 1.12-1.46) (Table 3). The seroprevalence rate in Heyuan was greater than that in Guangzhou (*OR*=1.82, *95% CI:* 1.57-2.11) (Table 3). In terms of the seroprevalence rates among different age groups, the 6–17 year age group had the lowest rate. Taking this group as the reference, the mumps seroprevalence rates were significantly greater in the following age brackets: 8 months-2 years, 3–5 years, 40–59 years, and 60 years or older (*OR*=4.76, 2.56, 1.74, and 2.78, respectively) (Table 3). The 8-month-2-year-old age group had the highest anti-mumps antibody titers and seroprevalence rates among all the age groups, with values of 32.21 NTUs (Fig 3A) and 88.75%, respectively (Table 2). Paired comparisons of age groups revealed statistically significant differences in the anti-mumps antibody titers between the < 8 months and 8 months-2 year age groups (*P* < 0.001) (Fig 3A and S1 Table), as well as between the 3–5 year and the < 8 months age groups (*P* < 0.001) (Fig 3A and S1 Table). The differences in the anti-mumps antibody titers between the age groups of <8 months and those aged 6–17 years, 18–39 years, 40–59 years, and>=60 years was not statistically significant (all *P* values >0.05) (Fig 3A and S1 Table).

**Table 3:** Results of multifactorial log-binomial regression models of seropositivity for IgG antibodies against mumps in healthy populations.

Model results		$\hat{\beta }$	*SE*	*Z*	*P-value*	*OR* ^*e(*^ *95%CI)*
Gender	Male					Reference
	Female	0.25	0.07	3.68	<0.001	1.28(1.12 ~ 1.46)
Age	<8ms^a^	0.08	0.20	0.41	0.684	1.08(0.74 ~ 1.59)
	8ms ~ 2ys^b^	1.56	0.38	4.14	<0.001	4.76(2.27 ~ 9.98)
	3 ~ 5 ys	0.94	0.17	5.41	<0.001	2.56(1.82 ~ 3.60)
	6 ~ 17 ys					Reference
	18 ~ 39 ys	0.18	0.16	1.11	0.267	1.20(0.87 ~ 1.65)
	40 ~ 59 ys	0.55	0.18	3.08	0.002	1.74(1.22 ~ 2.47)
	>=60 ys	0.93	0.18	5.18	<0.001	2.78(2.15 ~ 3.61)
Region	Guangzhou					Reference
	Heyuan	0.60	0.07	8.01	<0.001	1.82(1.57 ~ 2.11)
Year	2023					Reference
	2022	0.17	0.08	2.15	0.032	1.18(1.01 ~ 1.38)
	2021	0.51	0.09	5.94	<0.001	1.67(1.41 ~ 1.97)
Immunisation	No^c^					Reference
	Yes^d^	0.80	0.18	0.45	0.651	1.08(0.77 ~ 1.53)

^a^ms:months; ^b^ys:years; ^c^No: Including populations who have not been vaccinated or those with undisclosed information, with a total number of 4258. ^d^Yes: Including populations who received either 1 or 2 doses of vaccination, with a total number of 889; ^e^OR: Odds Ratio, it reflects the relationship between the exposure factor and the disease or outcome by comparing the odds of the event occurring in both groups.

### Trends of anti-mumps antibody titers with the vaccination program

There were 842 (61.06%) samples with an immunization history and 537 (38.94%) from the appropriate age group with no or unknown immunization history (Table 1). The mumps seroprevalence rates and anti-mumps antibody titers in the population with 1 dose or 2 doses (83.25%, 23.35 NTUs) of vaccine were both higher than those in the population with no vaccination history or an unclear vaccination history (73.18%, 19.66 NTUs) (*P* < 0.001) (Table 2). A mean decrease of 2.06% per year was observed in those who received 1 dose of vaccine, whereas a mean decrease of 10.33% per year was observed in those who received 2 doses of vaccine (S2 Table). According to the GAMs with temporal density weights, a healthy population would experience a decline to below the threshold after two doses of vaccine in approximately 12.3 years. A stratified analysis according to gender revealed that in the 1-dose-only population, the average annual declines were 2.80% for males and 0.74% for females. In the two-dose population, the average annual decline in males was 11.13%. In females, the average annual decrease was 9.25% (Fig 3C and S2 Table). In addition, we further processed the data for the 2-dose group by excluding samples beyond 36 months and refitting the model. The results mentioned above remain robust (S1 Fig).

### Relationship between vaccination procedures and anti-mumps antibody titers

The differences between the effects of vaccination procedures on mumps seroprevalence rates (*χ*^2^ = 70.47, *P* < 0.001) and anti-mumps antibody titers (*F* = 23.31, *P* < 0.001) were statistically significant (S3 Table). Overall, the mumps seroprevalence rates (*χ*^2^ = 23.26, *P* < 0.001) and anti-mumps antibody titers (*F* = 158.46, *P *< 0.001) were greater in the population that was vaccinated with 2 doses than in the population that was vaccinated with only 1 dose (S3 Table). The pairwise comparison revealed that the difference in anti-mumps antibody titers between those who received the AB vaccine and those who received the BA vaccine (i.e., the difference in anti-mumps antibody titers between those who received the AB vaccine and those who received the BA vaccine) was not statistically significant (*P* = 0.722) (Fig 3B and S4 Table). In contrast, the difference in anti-mumps antibody titers between those who received the BA vaccine and those who received the CC vaccine was statistically significant (*P* = 0.011) (Fig 3B and S4 Table). Furthermore, the differences in anti-mumps antibody titers among the other groups were not statistically significant (*P* > 0.05) (S4 Table).

## Discussion

The most recent data in China indicate that the number of mumps cases was 104,016 in 2022, representing approximately half of the global total, which remains among the highest in the world [18]. It is therefore evident that mumps causes a significant burden on China but is neglected compared with other infectious diseases, e.g., influenza. The WHO has recommended that MMR coverage should be greater than 95% to achieve herd immunity [19]. However, in this study, the seroprevalence rate for mumps IgG antibodies was 75.54%, which was similar to that reported in Luxembourg, Europe (75.40%) [20],but slightly lower than that reported in Luoyang, Henan Province (78.97%) [21] and comparable with that reported in Jiangxi Province (69.28%) [22], China, and was still far below the 90% immunity threshold that is required for the prevention of mumps [23], suggesting that the risk of mumps is high in the healthy population of Guangdong, as well as other provinces in China. Notably, there was a considerable discrepancy between the mumps seroprevalence rates (91.6%) in Guangdong Province in 2010 [24]. A study by Wang et al. revealed that the number of reported mumps outbreaks in 2020 was lowest in the historical records. However, rigorous preventive and control measures may be needed to prevent the spread of novel coronavirus infections [25], and the incidence of most infectious diseases during that period was lower than ever before or after. Therefore, effectively preventing mumps outbreaks is still necessary.

In our study, 74.40% of infants aged less than 8 months were positive for IgG antibodies to mumps. These data revealed the vertical transmission of IgG antibodies from mothers through the umbilical cord. Waalenborg et al. [26] reported that maternal mumps antibodies transferred in this manner disappear in infants at 6–9 months of age. The NIP also suggests reinforcing the protection of children under the age of 18 months from mumps. The mumps seroprevalence rate in the present study was 89.02% in children aged 8 months-2 years, which was close to 90%, indicating that the current 2-dose immunization program has almost achieved mumps immunity in this age group. In children aged 3–5 years, the mumps seroprevalence rate was 85.58%, which is slightly higher than that for children of the same age (79.0%) in Jiangsu Province [27]. An epidemiological study reported that the incidence of mumps was greater among children aged 3–9 years, with the majority of cases occurring in childcare facilities and primary schools [28]. The duration of protection from natural immunity is longer than that of vaccine-induced immunological protection. This is because vaccine-induced immunity is more rapidly attenuated by a lower frequency of memory B cells and a lower affinity for IgG [28,29]. Here, we observed lower seroprevalence rates in both the age groups of 6–17 years and 18–39 years (68.60% and 69.28%, respectively), which may be associated with the lower vaccination rates. Furthermore, primary vaccine failure may be a contributing factor to the sporadic outbreaks of mumps that have been observed [19]. Mumps infections have been shown to induce more than twice as much natural immunity as vaccine-induced immunity [30]. Nevertheless, the relationship between higher levels of natural infection-induced antibodies and enhanced protection remains to be further elucidated [31]. Overall, the antibody levels and seroprevalence rates were greater in females than in males (20.54 NTUs *vs.* 19.36 NTUs, 78.01% *vs.* 73.36%), which is consistent with the findings of previous studies. Li et al. reported antibody levels of 255.3 IU/mL in females and 235.5 IU/mL in males, with seroprevalence rates of 79.7% and 77.3%, respectively [12]. Similarly, Sun et al. reported antibody levels of 354.8 mIU/mL in females and 309.0 mIU/mL in males, with seroprevalence rates of 80.5% and 77.8%, respectively [27]. Stratified analysis revealed that the seroprevalence rate was higher in females than in males in most age groups. This could explain the higher incidence of infected males in mumps outbreaks [27].

The WHO has reported that the long-term protective efficacy of a single dose of mumps vaccine is only 60% to 90% [32]. The efficacy of the mumps vaccine also exhibits some variability, with estimates ranging from 79% to 95% [32]. Although the effectiveness of the mumps virus component of the MMR is not optimal, 2 doses of the vaccine ensure more than 15 years of immune protection compared with a single dose, which can prevent mumps outbreaks to some extent [30]. Previous studies have indicated that antibody titers decrease below the seropositivity threshold approximately 10–12 years after mumps vaccination [33,34]. On the basis of the serological data, we observed a similar decreasing tendency (approximately 12.3 years) of mumps IgG antibodies to the threshold in a population vaccinated with 2 doses, which is comparable with the classic cohort assay. Owing to limitations in this study, it is not yet possible to estimate precisely the protection period for the 1-dose population. In our study, we found an average annual decline of 10.33% in the population who received 2 doses of the vaccine, which, according to a study in the United States by Emma E. Seagle et al., represents an average annual decline of 9.2% for this group [33], and a study in Finland by Irja Davidkin et al. reported an average antibody decline rate after the second MMR of 9.9% [34].

We found that the antibody levels decreased more rapidly following the second dose of MMR. This observation is consistent with the results of other studies [31,34]. Additionally, we found that the antibody levels in males tended to decline more rapidly than in females in both the 1-dose population and the 2-dose population [35]. Concurrently, the differences in immunoprotection elicited by homologous and heterologous vaccinations, as well as those induced by heterologous sequential vaccinations, were not statistically significant. This may indicate that the immune responses that are induced by either vaccine are comparable. This may have implications for the adjustment of vaccination schedules. That is, if a particular MMR is lacking in supply for booster vaccinations, alternative use of other MMR could also achieve the goal of immune protection.

This study has several limitations that must be declared. First, there is currently no laboratory test that can accurately distinguish between antibodies that are induced by vaccination and those triggered by natural infection. However, in our study, we have also been unable to achieve this distinction. Second, since 2001, Guangdong Province has gradually established an immunization planning information system that covers the entire province. In this process, some vaccination information for the study subjects may be missing for historical reasons, which to some extent contributes to the lower vaccination rates we observed. Third, there was heterogeneity in the distribution of data in this study, especially in the group of females with 25–36 months between 2 doses of vaccine, where data were extremely limited. This phenomenon is mainly due to the bias in the enrolment process. However, to reduce the potential errors caused by this bias, we introduced time density weights into the GAMs to increase the robustness of the model. In the future, we will continue to expand the sample size of our research to increase its representativeness and universality, providing strong support for the formulation of more scientific and reasonable immunization strategies.

## Conclusion

The present study investigated the dynamics of the IgG levels of mumps antibodies through a cross-sectional serological study in a healthy population in Guangdong Province, China. The data indicated that a high level of mumps was endemic in the population without vaccination, whereas low serum levels of mumps IgG were detected in vaccinated participants, particularly individuals between the ages of 6–7 years. The most important finding is the faster declined tendency of antibodies in the vaccinated population; thus, booster vaccinations may be needed in adults. Worse, 73.18% of the unvaccinated population has mumps antibodies, which indicates that high levels of hidden mumps in China might be present, as well as in the remaining countries of the world.

## Methods

### Ethics statement

This study has been approved by the Medical Research Ethics Review Committee of Guangdong Provincial Center for Disease Control and Prevention (approval numbers: W96-027E-202018). Prior to the commencement of the study, formal consent was obtained in writing from all participants, including the parents or legal guardians of child participants, who signed the informed consent form.

### Subjects of the study

A cross-sectional study was performed from June 2021 to December 2023, a period of 2.5 years. A multistage stratified random sampling approach was used to select participants from Guangdong Province. First, 21 cities were categorized into two economic strata. Second, Guangzhou was selected from the high economic stratum, and Heyuan was selected from the low economic stratum (Fig 1). Finally, all participants were surveyed in one county and district. Hospitals or township health centers were selected as the survey units. The sample size was calculated via the following formula:


$$n=\frac{Z_{\alpha /2}^{2}(1-P)}{\delta^{2}P}$$

Here, *n* represents the sample size, the error probability *α* is set at 0.05, with the corresponding value of *Z*_*(α/2*)_ being 1.96, and the tolerance error *δ* is set to 0.05. *p* refers to the seroprevalence rate. Previous studies have indicated that the seroprevalence rate for mumps in the healthy population ranges from 70% to 80% [22,27]. On this basis, the *p* value was set to 70%, and the calculations indicated that at least 658 samples were needed per region. Since data collection was to be conducted over three consecutive years across two regions and considering participant compliance as well as other potential influencing factors, we decided to increase the sample size by 30%. A total of 5,152 participants finally interviewed, 5 participants with incomplete information were excluded, and finally, 5,147 participants were included in the study.

### Information collection

Basic information about the participants, including age, gender, residential address, and ID number, was collected through questionnaires. For children, this information was provided by their guardians. Information regarding vaccinations, such as vaccination dates, number of doses, vaccine types, and vaccination manufacturers, were retrieved from the Immunization Planning Information System (IPIS) of the Guangdong Provincial Center for Disease Control and Prevention by trained technicians. The healthy population was divided into seven age groups, namely, < 8 months, 8 months-2 years, 3–5 years, 6–17 years, 18–39 years, 40–59 years, and>=60 years. In addition, the study classified individuals with vaccination histories into six groups on the basis of the number of doses and manufacturers, namely, A, B, C, AB, BA, and CC. Among them, A, B, C represent Manufacturer A, B, and others for 1 dose vaccinations. AA, BB are 2 doses homologous vaccinations, CC stand for 2 doses others. AB and BA represent 2 doses heterologous vaccinations. These classifications were used in order to compare their impacts on the seropositivity rates and anti-mumps antibody titers and to explore the effects of sequential vaccinations on antibody levels.

### Serological antibody testing

Venous blood samples (3–5 mL) were taken from each participant, and the serum was separated and stored in a freezer at -70°C. An enzyme-linked immunosorbent assay (ELISA)(IBL International GmbH, Germany) was used to assess the concentration of mumps IgG antibodies in the serum samples. The experimental steps included, dispense standards/controls and samples, cover wells, incubate, wash wells, add conjugate, incubate, repeat washing, add TMB substrate solution, incubate, add stop solution and measure absorbance. The experimental procedure for ELISA was as per the instructions provided by the manufacturer. All ELISA plates were evaluated using an enzyme-labeled instrument (BioTek Epoch, Winooski, VT, USA). The absorbances of all the wells were measured at 450 nm, and the absorbance values for each standard/control and sample in the plate layout were recorded. In accordance with the instructions of the ELISA kit, the final values represent the anti-mumps antibody titers, which are expressed in “NovaTec units”. The formula is as follows,


$$\frac{Sample\;(mean)absorbance\;value*10}{Cut-off}=NovaTec\;Units(NTU)$$

Here, the sample (mean) absorbance value is directly measured. During the experiment, each ELISA plate included two wells for measuring the absorbance value of the “Cut-off Control”. The “Cut-off ” in the formula is the mean absorbance value of the “Cut-off Control” determinations. The anti-mumps antibody titers of more than 11 NTUs is considered positive, whereas a result of 9–11 NTUs is considered equivocal. Values less than 9 NTUs are considered negative. If antibodies against the pathogen cannot be detected clearly, it is recommended that the test be repeated. If the results are equivocal again, then the sample is judged as negative. The presence of IgG antibodies indicated a history of prior infection or successful vaccination.

### Data analysis

Statistical software. The statistical analyses were conducted via Stata.17 (StataCorp LLC, USA) and R 4.3.2.

Principle of analysis. The two-sided test level was set at α = 0.05. A *p* value of less than 0.05 indicates that the observed difference is statistically significant. All the statistical inferences were made via two-sided tests, and 95% confidence intervals were employed for estimating the confidence intervals of the parameters. As far as possible, parametric tests were employed. In instances where the data did not meet the conditions of parametric tests, data conversion methods could be used to make them meet the conditions. If this was unsuccessful, nonparametric tests were employed. Missing value processing. Multiple Imputation by Chained Equations (MICE) based on a random forest model was used to interpolate the data.

Descriptive statistics. The mean values are provided for measurement information. The frequencies and corresponding percentages are provided for the count data. The measured data were subjected to t tests and Analysis of Variance (ANOVA), with approximate t tests and Kruskal‒Wallis tests employed when the variance was not uniform. The count data were tested by *χ*^2^. The decline in IgG antibody levels over time was modeled via generalized additive models (GAMs) and the threshold was set at 11 NTUs to ensure the sensitivity and reliability of the test. Finally, in the GAMs, we introduced temporal density weights to predict the time when the population’s antibody levels reach the threshold.

## Supporting information

S1 Fig Trends in anti-mumps IgG attenuation in 1-dose and 2-dose vaccine groups after excluding samples vaccinated for >192 months and >36 months, respectively. The solid line represents the fitted declining trend of IgG antibodies against mumps, and the shaded area represents the *95%CI*. The dashed line represents a threshold of 11 NTUs.
(TIF)

S1 Table Pairwise comparisons of the individuals’ anti-mumps antibody titers between age groups. ^a^ms:months; ^b^ys:years.
(DOCX)

S2 Table The extent of antibody decline in the vaccinated population and the estimated time to reach the threshold. (DOCX)

S3 Table Comparison of the impact of vaccination and vaccination procedures on mumps seropositivity(%) and anti-mumps antibody titers (NTUs). ^a^vaccination manufacturer: A, B, C represent Manufacturer A, B, and others for 1 dose vaccinations. AA, BB are 2 doses homologous vaccinations, CC stand for 2 doses others. AB and BA represent 2 doses heterologous vaccinations.
(DOCX)

S4 Table Pairwise comparisons of the individuals’ anti-mumps antibody titers between vaccination schedules. ^a^Group: A, B, C represent Manufacturer A, B, and others for 1 dose vaccinations. AA, BB are 2 doses homologous vaccinations, CC stand for 2 doses others. AB and BA represent 2 doses heterologous vaccinations.
(DOCX)

S1 Data The numerical data used in this study. (XLSX)
