# Supplementary figures and images for: Decade-long protection of the mumps vaccine: Insights from a large-scale serological study
Source: PLoS Negl Trop Dis. 2025 Jun 3;19(6):e0013125. doi: 10.1371/journal.pntd.0013125 (PMC12165342; doi:10.1371/journal.pntd.0013125)

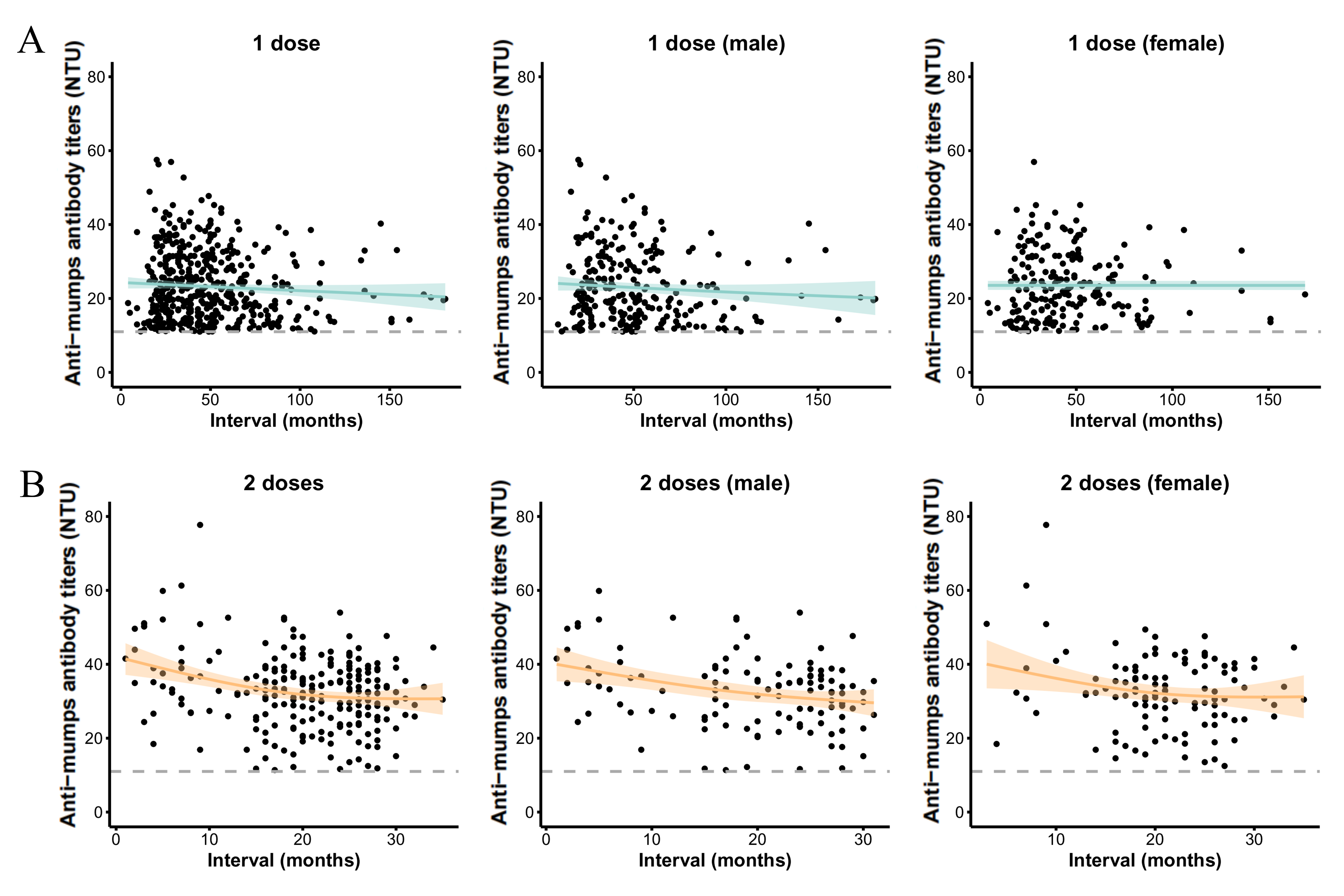

Supplement: S1 Fig — The solid line represents the fitted declining trend of IgG antibodies against mumps, and the shaded area represents the 95%CI. The dashed line represents a threshold of 11 NTUs. (TIF) [file pntd.0013125.s001.tif]
