# Supplementary material for: Decade-long protection of the mumps vaccine: Insights from a large-scale serological study
Source: PLoS Negl Trop Dis. 2025 Jun 3;19(6):e0013125. doi: 10.1371/journal.pntd.0013125 (PMC12165342; doi:10.1371/journal.pntd.0013125)
Supplement: S1 Table — ams:months; bys:years. (DOCX) [file pntd.0013125.s002.docx]

**Supplemental Table 1. Pairwise comparisons of the individuals' anti-mumps antibody titers between age groups.**

| Group | 0~ | 8ms~ | 3ys~ | 6ys~ | 18ys~ | 40ys~ |
| --- | --- | --- | --- | --- | --- | --- |
| 8ms^a^~ | 13.32  <0.001 |  |  |  |  |  |
| 3ys^b^~ | 4.90  <0.001 | -8.43  <0.001 |  |  |  |  |
| 6ys~ | -1.52  1.000 | -14.85  <0.001 | -6.42  <0.001 |  |  |  |
| 18ys~ | -0.98  1.000 | -14.30  <0.001 | -5.87  <0.001 | 0.55  1.000 |  |  |
| 40ys~ | 0.24  1.000 | -13.09  <0.001 | -4.67  <0.001 | 1.76  0.215 | 1.21  0.156 |  |
| >=60ys | 1.34  0.754 | -11.98  <0.001 | -3.33  <0.001 | 2.87  <0.001 | 2.32  <0.001 | 1.11  0.666 |

^a^ms:months; ^b^ys:years;
