# Supplementary material for: Decade-long protection of the mumps vaccine: Insights from a large-scale serological study
Source: PLoS Negl Trop Dis. 2025 Jun 3;19(6):e0013125. doi: 10.1371/journal.pntd.0013125 (PMC12165342; doi:10.1371/journal.pntd.0013125)
Supplement: S2 Table — (DOCX) [file pntd.0013125.s003.docx]

**Supplemental Table 2. The extent of antibody decline in the vaccinated population and the estimated time to reach the threshold.**

|  | Decrease per year (%) | Estimated time to reach the threshold (years) |
| --- | --- | --- |
| 1dose (total) | -2.06 | -- |
| 1dose (male) | -2.80 | -- |
| 1dose (female) | -0.74 | -- |
| 2doses (total) | -10.33 | 12.3 |
| 2doses (male) | -11.13 | -- |
| 2doses (female) | -9.25 | -- |
