# Supplementary material for: Decade-long protection of the mumps vaccine: Insights from a large-scale serological study
Source: PLoS Negl Trop Dis. 2025 Jun 3;19(6):e0013125. doi: 10.1371/journal.pntd.0013125 (PMC12165342; doi:10.1371/journal.pntd.0013125)
Supplement: S3 Table — avaccination manufacturer: A, B, C represent Manufacturer A, B, and others for 1 dose vaccinations. AA, BB are 2 doses homologous vaccinations, CC stand for 2 doses others. AB and BA represent 2 doses heterologous vaccinations. (DOCX) [file pntd.0013125.s004.docx]

**Supplemental Table 3. Comparison of the impact of vaccination and vaccination procedures on mumps seropositivity(%) and anti-mumps antibody titers (NTUs)**

| Characteristics |  | Seropositivity  (%) | *χ^2^* | *P-value* | anti-mumps antibody titres (NTUs) | *t/F* | *P-value* |
| --- | --- | --- | --- | --- | --- | --- | --- |
|  |  |  |  |  |  |  |  |
| vaccination manufacturer^a^ | A | 72.73 | 70.47 | <0.001 | 21.47 | 23.31 | <0.001 |
|  | AA | 100 |  |  | 32.73 |  |  |
|  | AB | 87.5 |  |  | 30.24 |  | <0.001 |
|  | B | 83.09 |  |  | 24.16 |  |  |
|  | BA | 90.91 |  |  | 35.67 |  | <0.001 |
|  | BB | 95.4 |  |  | 31.90 |  |  |
|  | C | 63.58 |  |  | 20.85 |  |  |
|  | CC | 64.29 |  |  | 20.59 |  |  |
| vaccination | 1 dose | 77.18 | 23.26 | <0.001 | 19.69 | 158.46 | <0.001 |
|  | 2 doses | 90.71 |  |  | 29.85 |  |  |

^a^vaccination manufacturer: A, B, C represent Manufacturer A, B, and others for 1 dose vaccinations. AA, BB are 2 doses homologous vaccinations, CC stand for 2 doses others. AB and BA represent 2 doses heterologous vaccinations.
