# Supplementary material for: Decade-long protection of the mumps vaccine: Insights from a large-scale serological study
Source: PLoS Negl Trop Dis. 2025 Jun 3;19(6):e0013125. doi: 10.1371/journal.pntd.0013125 (PMC12165342; doi:10.1371/journal.pntd.0013125)
Supplement: S4 Table — aGroup: A, B, C represent Manufacturer A, B, and others for 1 dose vaccinations. AA, BB are 2 doses homologous vaccinations, CC stand for 2 doses others. AB and BA represent 2 doses heterologous vaccinations. (DOCX) [file pntd.0013125.s005.docx]

**Supplemental Table 4. Pairwise comparisons of the individuals' anti-mumps antibody titers between vaccination schedules.**

| vaccination manufacturer^a^ | A | AA | AB | B | BA | BB | C |
| --- | --- | --- | --- | --- | --- | --- | --- |
| AA | 11.26  0.618 |  |  |  |  |  |  |
| AB | 8.77  0.358 | -2.49  1.000 |  |  |  |  |  |
| B | 2.69  0.990 | -8.57  0.779 | -6.08  0.325 |  |  |  |  |
| BA | 14.20  0.001 | 2.94  1.000 | 5.427  0.722 | 11.51  <0.001 |  |  |  |
| BB | 10.43  0.015 | -0.83  1.000 | 1.65  0.999 | 7.74  <0.001 | -3.77  0.651 |  |  |
| C | -0.62  1.000 | -11.88  0.383 | -9.39  0.017 | -3.57  0.990 | -15.08  0.011 | -11.05  <0.001 |  |
| CC | -0.88  1.000 | -12.14  0.630 | -9.66  0.484 | -3.57  0.990 | -15.08  0.011 | -11.31  0.100 | -0.26  1.000 |

^a^vaccination manufacturer: A, B, C represent Manufacturer A, B, and others for 1 dose vaccinations. AA, BB are 2 doses homologous vaccinations, CC stand for 2 doses others. AB and BA represent 2 doses heterologous vaccinations.
